# Comparative Effect of Cricket Protein Powder and Soy Protein Isolate on Gel Properties of Indian Mackerel Surimi

**DOI:** 10.3390/foods11213445

**Published:** 2022-10-30

**Authors:** Panumas Somjid, Worawan Panpipat, Ling-Zhi Cheong, Manat Chaijan

**Affiliations:** 1 Food Technology and Innovation Research Center of Excellence, School of Agricultural Technology and Food Industry, Walailak University, Nakhon Si Thammarat 80160, Thailand; 2 Zhejiang-Malaysia Joint Research Laboratory for Agricultural Product Processing and Nutrition, College of Food and Pharmaceutical Science, Ningbo University, Ningbo 315211, China

**Keywords:** protein additive, soy protein isolate, cricket powder, mackerel, surimi, gel

## Abstract

This work comparatively investigated the effects of different levels (0, 1, 3, and 5%, *w/w*) of cricket protein powder (CP) and soy protein isolate (SPI) on the gel properties of mackerel surimi. Both SPI and CP enhanced the rheological properties of surimi pastes during heating, as indicated by the increase in G′ and G″ and the decrease in tan δ. With increasing SPI content, the proteolytic inhibition, gel properties, water-holding capacity, and textural profiles of surimi gel were markedly enhanced. Molecular driving-force results showed that SPI markedly promoted the hydrophobic interaction, while disulfide bonds were dominant in CP-added gel. However, the whiteness of surimi gels tended to decrease with the increased levels of both additives, in particular CP. Sodium dodecyl sulfate-polyacrylamide gel electrophoresis (SDS-PAGE) demonstrated that SPI hindered the polymerization of myosin heavy chain while CP participated in the formation of non-disulfide covalent bonds with actin. Fourier transform infrared (FTIR) spectra indicated that CP and SPI did not influence the secondary structure of proteins in surimi. Scanning electron microscopy (SEM) demonstrated that CP or SPI induced the myofibrillar protein to form smoother and compact gel network structures. Overall acceptability of the mackerel surimi gel can be improved by the incorporation of 5% SPI while CP had a negative impact on several parameters. However, CP showed the remarkable ability to prevent the lipid oxidation of the gel after storage at 4 °C for 7 days. Overall, both SPI and CP demonstrated positive impacts on the gelling characteristics of mackerel surimi; however, SPI was more advantageous than CP in terms of the gel-strengthening effect and sensory qualities. This study offered a potential use for plant and insect proteins as functional and nutritional ingredients for the production of dark-fleshed fish surimi.

## 1. Introduction

Surimi, a ground and water-leached fish muscle paste, is an intermediate material used in the production of gel-based products and imitation seafood products, such as fish balls, fish tofu, chikuwa, and imitation crab sticks. For the production of surimi in Thailand, several species of white-fleshed fish, including threadfin bream (*Nemipterus* spp.), bigeye snapper (*Priacanthus* spp.), goatfish (*Upeneus* spp.), and lizardfish (*Saurida* spp.) have been popular [1]. However, the population of these fish has occasionally been uncertain and insufficient as a result of the season and over-catching [2]. Hence, utilizing the availability of alternative resources is necessary for surimi manufacturing, such as small fatty pelagic fish species (dark-fleshed fish species). Stocks of sardine and mackerel, two underutilized fish species, are plentiful and steady in Southern Thailand. Indian mackerel (*Rastrelliger kanagurta*) is one type of mackerel that has a high value-added potential to fill the market’s primary fish-resource need [3,4]. Unfortunately, problems with the color and gel-forming properties associated with high lipid levels and a high content of sarcoplasmic proteins consequently resulted in a degree that would restrict its use [5].

To address such a drawback, many novel attempts have been made to improve the gel properties. For example, electron irradiation [6], ultrasound-assisted process [7], high hydrostatic pressure [8], and microwave heating [9]. These methods, however, are frequently challenging and costly. Adding exogenous additives into surimi is also another choice of surimi research and is gaining more and more attention to enhance the gel properties. Numerous food-grade ingredients have been incorporated, such as whey protein concentrate [10], modified starch [11], egg-white protein [12], phenolic compounds [13], olive leaf powder [14], transglutaminase [15], chitosan [16], and hydrocolloids [17,18,19]. However, the addition of these ingredients may have a detrimental effect on the surimi gel, particularly on the development of an off-flavor or discoloration [20]. It is essential to research innovative methods for manufacturing high-quality surimi-based products in order to address the rising demand for these products due to their distinctive textural qualities and high nutritive value.

Among farmed insects, the house cricket (*Acheta domesticus*) is one of the most promising species for human consumption due to its nutritional value, safety for consumption, and lack of side effects. It is also an environmentally friendly alternative to meat consumption and a good source of protein (up to approximately 70% of dry matter) [21,22]. This enhances functioning in food formulations and, as an alternative protein, making them acceptable for a variety of food products [23]. Unfortunately, some consumers are reluctant to accept insects as food. Additionally, it is challenging to grow these insects on an industrial scale because the consumption of edible insects is avoided owing to the aversion to their appearance [24]. One possibility is to incorporate edible insects into familiar foods so that the insects are no longer recognizable [25,26]. As a result, it is important to turn crickets into cricket powder (CP) in order to become acceptable. Recently, CP has been paid more attention for being incorporated into other foods such as bread [27,28], biscuits [29,30], and pasta [31]. However, to our knowledge, there is presently no documentation on the use of insects incorporated into gelled products on their gel properties and the utilization of CP as a food ingredient is still relatively sparse and hence poses functionality challenges in food production.

Soy protein isolate (SPI) has been commonly applied in food processing due to its high nutritional content, techno-functional properties, and reasonable price [32]. SPI contains a high amount of protein (at least 90% of dry matter) and can improve the gel strength of the surimi products by forming a dense 3D-network structure resulting from the association between the SPI and muscle proteins [33]. The endogenous protease inhibitor found in SPI can prevent gel weakening, or “modori” and improve the texture of surimi [34]. According to Park [35], SPI at 1 % (*w/w*) increased the gel strength in medium-grade Alaska Pollock surimi. Luo et al. [34] discovered that SPI 10% (*w/w*) might reduce the occurrence of modori in silver carp surimi. Although SPI has been applied in surimi products, the results and conclusions from various studies have not been consistent. Moreover, the application of SPI in surimi gel from dark-fleshed fish has yet to be performed, and its comparative evaluation with other sources of protein additives on the gel properties should be investigated.

Therefore, the objective of this research was to compare the effects of different amounts (1%, 3%, and 5% (*w/w*)) of CP and SPI incorporated into low-grade Indian mackerel surimi on physicochemical properties, rheological behavior, textural characteristics, and the acceptability of the resulting gel. This would reveal a more thorough understanding of the interactions between CP/SPI and mackerel surimi and their effects on gel properties. Additionally, it would also offer a conceptual foundation for the sensible application of natural exogenous protein for enhancing the textural and nutritional aspects of surimi products.

## 2. Materials and Methods

### 2.1. Materials

The protein powders used in the surimi formulations: soy protein isolate (SPI) (protein content 90%) were purchased from PTK Solution and Supplies Ltd. (Bangkok, Thailand) and cricket powder (CP) (protein content 80%) was procured from JR Unique Foods Ltd. (Udon Thani, Thailand).

The Indian mackerel (*R. kanagurta*) used for preparing surimi, with an average weight of 40–50 g, were purchased from the local market in Thasala, Nakhon Si Thammarat, Southern Thailand. The fish were packed in ice with a fish per ice ratio of 1:2 (*w/w*), and transported to the laboratory within 30 min. The fish were washed, filleted, and skinned immediately. The whole muscles were minced homogeneously in a meat grinder (Panasonic MK-G20NR-W, Osaka, Japan).

### 2.2. Preparation of Surimi

Two washing cycles for Indian mackerel surimi production were prepared according to the method of Somjid et al. [2]. With a 3:1 (*v/w*) water to mince ratio, the fish mince was rinsed in cold (4 °C) water. The mixture was stirred manually for 10 min in iced basket and allowed to settle for 10 min. After being filtered through a nylon screen layer, the slurry was washed for a second time employing the same procedure as the first cycle. Then, the excess water was manually squeezed out and centrifuged at 2500× *g* for 10 min using Sorvall Legend XTR Centrifuge (Thermo Fisher Scientific Inc., Yokohama, Japan). The washed mince was mixed for 1 min with the addition of sorbitol and sucrose, each at 4% *w/w*, as cryoprotectants using a Mara food processor (model 2102240; Taipei, Taiwan) and the moisture content was adjusted to an average of 80%. The fresh surimi was packed, 100 g each, into low-density poly-ethylene bags. Finally, the samples were frozen and stored at −18 °C before being used. The period of frozen storage was no longer than a month.

### 2.3. Preparation of Surimi Gel Added with Protein Additives

Frozen Indian mackerel surimi was thawed at 4 °C for 12 h. Then, the thawed surimi was blended with 2.5% (*w/w*) NaCl for 1 min to form the paste, using a Mara food processor. The surimi paste was then blended with protein additive powder (CP or SPI) at various levels of 1%, 3%, and 5% (*w/w*) and chopped for an additional 5 min. Due to the brown color of both CP and SPI, they can interfere with the surimi gel’s whiteness at high concentrations. According to the preliminary study, surimi gel’s color was negatively impacted by a level greater than 5%. Therefore, it was decided to keep the maximum concentration of each protein additive at 5%. The control (no additive) was also created by the same procedure. Surimi pastes were taken for the oscillatory dynamic rheology test prior to the preparation of the thermally induced gel (see Section 2.4). To prepare the gel, the surimi pastes were stuffed into a casing (polyvinylidene chloride/a diameter of 2.5 cm) and both ends were firmly tied. The surimi gels were produced by setting at 40 °C for 30 min and followed by cooking at 90 °C for 20 min in a water bath. The properties of the gels were examined after being chilled in ice water for 30 min and kept at 4 °C overnight [2].

### 2.4. Oscillatory Dynamic Rheology

The dynamic rheology of the surimi pastes was measured according to the method of Somjid et al. [2], using a Rheometer (HAAKE MARS 60; Thermo Fisher Scientific Inc., Yokohama, Japan). A total of 0.5 g of the surimi paste was spread out on the sample holder, which was set up with a 35 mm parallel plate geometry A thin coating of oil was added to the dehydration process, and the space between the plate and the sample holder was fixed at 0.5 mm. The temperature sweep mode was used to monitor the changes in rheological indices, including elastic modulus (G′), viscous modulus (G″), and tan δ during heating at a constant frequency of 1 Hz and an amplitude strain of 2%. A 3 °C/min heating rate was used to increase the temperature sweep from 20 °C to 90 °C.

### 2.5. Trichloroacetic acid (TCA)-Soluble Peptide

Chopped gel was mixed with 9 volumes of 5% TCA and homogenized for 2 min at 11,000 rpm (IKA Labortechnik homogenizer, Selangor, Malaysia). Then, it was incubated at 4 °C for 1 h followed by centrifugation at 8000× *g* for 5 min, using an RC-5B plus centrifuge (Sorvall, Norwalk, CT, USA). TCA-soluble peptides in the supernatant were determined by the Lowry method [36] and reported as µmol/tyrosine/g sample [37].

### 2.6. Breaking Force, Deformation, Gel Strength and Texture Profile Analysis (TPA)

A texture analyzer (LR 5K; LLOYD Instruments, West Sussex, UK) was used to evaluate the textural properties of surimi gels. The load cell capacity was 5 kg and the return distance was 35 mm. The gels were placed at room temperature (27–29 °C) until equilibrated for 1 h before analysis and then cut to cylinders (2 × 2.5 cm^2^). The breaking force (g) and deformation (mm) were measured using a spherical probe (diameter of 5 mm/test speed of 1.0 mm/s/trigger force of 0.05 N) which was applied on the gel surface until a fracture formed in it. In order to calculate gel strength (g·mm), breaking force (g), and deformation (mm) were multiplied [2].

The TPA of surimi gels was subjected to two-cycle compression using a texture analyzer with a cylindrical aluminum probe (diameter 70 mm) at a test speed of 1.0 mm/s (50% of original gel height) and a trigger force of 0.05 N. The rates before and after the measurement were both set at 1.00 mm/s. The time interval between the first and the second compressions was 10 s. The rate of data acquisition was 400 pps. In order to exclude out the impact of low temperatures, samples were maintained at room temperature before the experiment [2]. 

### 2.7. Expressible Moisture Content (EMC)

The EMC was determined by the method of Chaijan et al. [38]. Gel sample (5 mm thicknesses) was weighed (W1). The sample was inserted between Whatman paper No. 1 (2 pieces) on the top and 3 pieces at the bottom. A standard mass (5 kg) was placed on the sample and held for 2 min. Then, the compressed sample was weighed again (W2). The EMC was estimated and reported as percentage of sample weight:

EMC (%) = [(W1 − W2)/W1] × 100(1)

### 2.8. Whiteness

Color measurement for surimi gel was determined by the method of Panpipat and Chaijan [39]. The gel color (L*, a*, and b*) was determined using a Hunterlab Miniscan/EX instrument (10° standard observers, illuminant D65; Hunter Assoc. Laboratory, VA, USA) and the whiteness was calculated using the following equation:

(2) $$\mathrm{Whiteness}=100\;–\sqrt{{\left(100-L*\right)}^{2}+{a*}^{2}+{b*}^{2}}$$

where L* is lightness; a* is redness/greenness; b* is yellowness/blueness.

### 2.9. Molecular Driving Forces

The chemical interactions between the proteins in the gels were determined by the method of Mi et al. [32]. Different chemical reagents that were chosen for their ability to cleave particular types of interactions were applied to gels. Chopped gels (2 g) were homogenized at 12,000 rpm for 1 min with 10 mL each of SA (0.05 M NaCl), SB (0.6 M NaCl), SC (0.6 M NaCl + 1.5 M urea), SD (0.6 M NaCl + 8 M urea), and SE (0.6 M NaCl + 8 M urea + 0.05 M β-mercaptoethanol (βME)) individually. The homogenate was stored at 4 °C for 1 h and then centrifuged at 10,000× *g* for 15 min. The supernatant was collected to determine the protein content by the Biuret method [40]. The index of those bonds were estimated as the following equation:

Index of ionic bonds = SB − SA(3)

Index of hydrogen bonds = SC − SB(4)

Index of hydrophobic interactions = SD − SC(5)

Index of disulfide bonds = SE − SD(6)

### 2.10. Sodium Dodecyl Sulfate-Polyacrylamide Gel Electrophoresis (SDS-PAGE)

Protein patterns of all samples were studied using SDS-PAGE [41]. Samples were solubilized using a 5% SDS solution (90 °C). Protein content of each sample was then adjusted to 10 mg/mL using the Biuret method [40] and mixed with sample buffer under non-reducing and reducing conditions. The samples were boiled (85 °C/5 min) before being loaded into stacking and separating gels made by 4% (*w/v*) and 10% (*w/v*) acrylamide, respectively. Then, electrophoresis at a 15 mA/gel was applied (Mini Protein II unit; Bio-Rad Laboratories, Inc., Richmond, CA, USA). Standard markers were used. The gel was stained for 30 min with Coomassie Brilliant Blue R250, after which it was discolored for 12 h by a methanol and acetic acid solution.

### 2.11. Fourier Transform Infrared (FTIR) Spectra

A Bruker INVENIO-S FTIR spectrometer (Bruker Co., Ettlingen, Germany) fitted with an attenuated total reflection (ATR) diamond crystal cell was used to capture the FTIR spectra of gels. Using a freeze-dryer (CoolSafe 55; ScanLaf A/S, Lynge, Denmark), the samples were dried to the solid state. Thirty-two scans with a resolution of 4 cm^−1^ were used to determine the IR absorption in the range of 500–4000 cm^−1^. The data were examined using the program OPUS 8.5. (Bruker Optik GmbH 2020, Ettlingen, Germany).

### 2.12. Scanning Electron Microscopy (SEM) 

According to Somjid et al. [42], the microstructure of gels was observed using SEM (GeminiSEM; Carl Ziess Microscopy, Germany). The gels were cut into 1–2 mm cubes, fixed with 2.5% (*v*/*v*) glutaraldehyde in 0.2 M phosphate buffer (pH 7.2), and washed with 0.1 M phosphate buffer and distilled water, respectively, for 2 h at room temperature. The fixed samples were subsequently dehydrated in ethanol for 10 min at serial concentrations of 50%, 70%, 80%, 90%, and 100%. After critical point drying with CO_2_ as the transition fluid, dried samples were mounted on a bronze stub and given a gold sputter coating. An SEM was used to scan the samples with an acceleration voltage of 3 kV with 10,000× magnification.

### 2.13. Sensory Property 

Sensory analysis of surimi gel was performed using 9-point hedonic scale [43]. In order to evaluate surimi gels for appearance, color, odor, texture, taste, and overall likeness, 50 untrained panelists (aged 20 to 45) were recruited. Gel samples were served at room temperature under fluorescent daylight-style illumination in a covered white plastic cup. The panelists were asked to rinse their mouths with drinking water at room temperature between the samples. The experiment was approved by the Human Research Ethics Committee of Walailak University (WUEC-21-125-01).

### 2.14. Thiobarbituric Acid Reactive Substances (TBARS)

Chopped gels were divided into 2 groups and kept at 4 °C in a plastic cup with a lid for analyzing TBARS on Days 0 and 7, using the method described by Buege and Aust [44]. TBARS content was recorded as mg malondialdehyde (MDA) equivalent/kg sample.

### 2.15. Statistical Analysis

Analysis of variance (ANOVA) was used to analyze the data. Duncan’s multiple-range test was used to compare the means. SPSS 16.0 for Windows was used to conduct the statistical analysis (SPSS Inc., Chicago, IL, USA). All experimental data were reported by mean ± standard deviation.

## 3. Results and Discussion

### 3.1. Dynamic Rheological Properties

**Figure 1:**
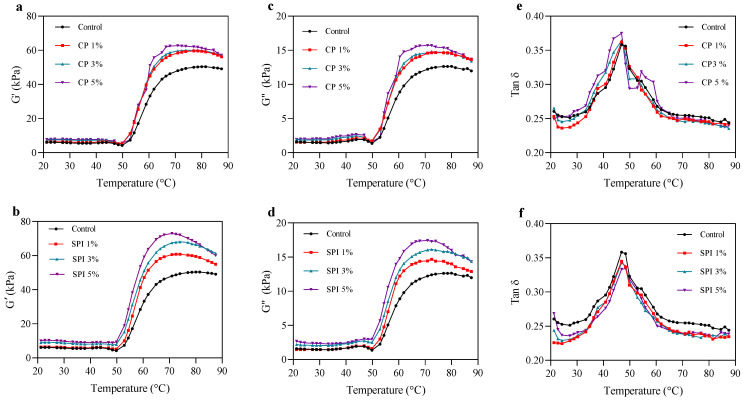
Changes in storage modulus (G′), loss modulus (G″), and loss tangent (tan δ) of mackerel surimi gels at various concentrations of cricket powder (CP) (**a**,**c**,**e**) and soy protein isolate (SPI) (**b**,**d**,**f**) during the heating.

The dynamic rheology test is widely used to observe the viscoelastic behavior and molecular interactions of surimi during gelation, which is the basis for texture development [45]. The rheological properties of the surimi paste added with and without CP and SPI during heating from 20 °C to 90 °C are shown in Figure 1. Regardless of the amount of additive protein present, the patterns of the storage modulus (G′) and loss modulus (G″) were comparable. Generally, the G′ is always higher than the G″, which means that surimi pastes have greater elastic than viscous tendencies, exhibiting the usual viscoelastic solid-like characteristics of a gel network [46]. As seen in Figure 1a–d, all paste samples exhibited the similar trend of G′ and G″ curves during the temperature sweep. The gradual increase in G′ and G″ from around 35 °C to approximately 47 °C revealed the unfolding of heavy meromyosin at low temperatures and allowed the cross-linking between myosin filaments via hydrogen bonds, which formed a weak protein network and an increase in the viscoelasticity of the sample [47,48]. Subsequently, the drop in G′ and G″ was noticeable as the temperature increased from 40 °C to 50 °C, which may possibly result from the helix-to-coil transformation of light meromyosin. The partial breakdown of hydrogen bonds might be caused by heating, which can disrupt the formation of protein networks already formed [49]. Furthermore, another vital reason was due to the degradation of the actin–myosin network structure mediated by endogenous proteolytic enzymes, whose optimal activity was reported in the temperature range of 50–60 °C [50]. Subsequently, it led to a large increase in fluidity and protein mobility, resulting in a temporary decline in G′ [51]. The second increase in G′ and G″ with increasing temperature occurred until 70 °C. At this stage, higher attractive forces (such as disulfide bond and hydrophobic interaction) caused by an increase in the cross-linking of dissociated proteins and the denaturation of myosin heavy chain and actomyosin led to the formation of thermo-irreversible gel networks [52]. Reactive groups or domains in unfolded proteins may promote interaction aggregation. According to Balange and Benjakul [13], hydrophobic domains may have interacted via hydrophobic–hydrophobic interaction, and sulfhydryl groups could be oxidized, leading to the generation of a disulfide bond. As a result, protein aggregation occurs. After that, the G′ and G″ were once more decreased till the temperature approached 90 °C. This could be due to hydrogen bonding destruction at high temperatures [51].

An onset of gelation or the development of an elastic protein network was indicated by an increase in G′ [53]. The addition of CP or SPI significantly increased the G′ value. Specifically, as the SPI level increased, the G′ value of the entire system similarly increased. The initial G′ of the samples containing CP and SPI for all levels was higher than the control. Adding CP and SPI to surimi led to an increase in the protein level and a decrease in the moisture content of the gel system. This led to an increase in the viscosity of the surimi paste, especially with the integration of SPI, as a result of the development of hydrogen bonds and the interaction of ionic bonds between added protein and myofibrils in surimi. The presence of CP and SPI at the highest level exhibited the highest G′ values of 15,708 and 17,435 Pa, respectively, compared to the control (12,222 Pa). The study revealed that myofibrils and protein additives were cross-linked, with the SPI–myofibril interaction being more potent and exhibiting a more ordered gel network structure than the CP–myofibril association. It is possible that during heating, more reactive groups, particularly hydrophobic ones, in the SPI structure were exposed, increasing the augment contact and resulting in a final gel structure that was stronger and more elastic. This suggests that the influence of SPI on surimi gelation may vary according to the extent of its denaturation or aggregation.

**Figure 2:**
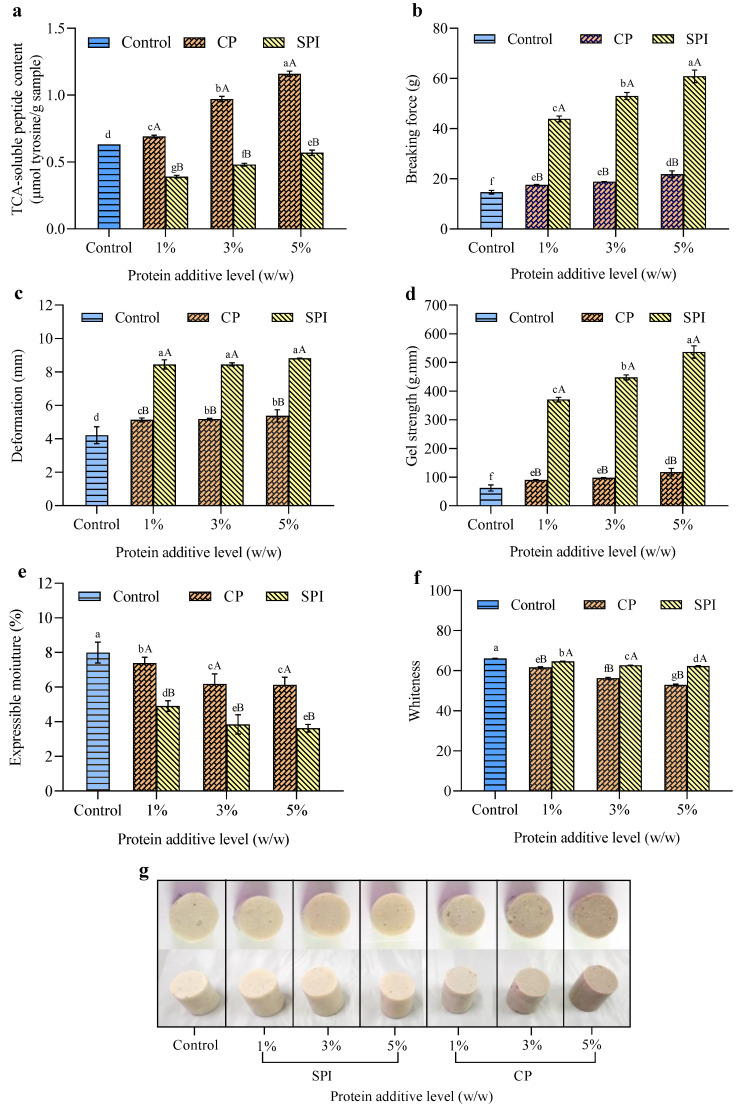
Effects of the addition of cricket powder (CP) and soy protein isolate (SPI) at different concentrations on TCA-soluble peptide (**a**), breaking force (**b**), deformation (**c**), gel strength (**d**), expressible moisture (**e**), whiteness (**f**), and appearance (**g**) of mackerel surimi gels. Bars represent standard deviation from triplicate determinations. Different uppercase letters between additives at the same level and different lowercase letters in all samples indicate significant differences (*p* < 0.05).

Both CP and SPI reduced the tan δ of the final phase and the significant difference was found in SPI added, compared to the control (Figure 1e,f). Based on the greater strength of the intermolecular connections in the gel matrix, the network becomes more cohesive as tan δ decreases [54]. The sample is a pure solid if tan δ = 0, and a pure liquid if tan δ = ∞ [55]. The tan δ of CP and SPI was less than 1.0 and lower than the control. With the addition of CP and SPI, the samples behaved more like an elastic fluid and displayed enhanced gelation characteristics. Based on the findings, it appears that CP or SPI contribute to the development of the gel network. The high G′ values indicated the enhancement of the surimi gel properties and the low tan δ values referred to a highly elastic network structure, which is consistent with the gel strength displayed in Figure 2.

### 3.2. Gel Properties

The dynamic rheological test offers an observational viewpoint on the interactions among CP–surimi and SPI–surimi during gel formation. In order to more clearly demonstrate the impacts of CP and SPI on the gel properties of mackerel surimi, TCA-soluble peptide, breaking force, deformation, gel strength, EMC, and whiteness were performed on the gel samples as shown in Figure 2.

#### 3.2.1. TCA-Soluble Peptide Content

The content of small peptides in surimi and the proteolytic degradation that occurs during the heat treatment of surimi gels are reflected in the TCA-soluble peptides [50]. The residual protease, such as the myofibril-bound protease in the surimi, can typically be activated during thermal gelation, especially at 50–70 °C, which has a major impact on the strength of the surimi gel [56]. Furthermore, Kudre et al. [57] reported that sardine surimi’s proteolysis might be accelerated at 40 °C and peak at 65 °C. According to the findings, the TCA-soluble peptide content of the gel added with CP tended to increase significantly with an increasing level of CP in comparison to the control (*p* < 0.05) (Figure 2a). The residual proteinase enzyme in the CP may be activated during thermal gelation, causing more myofibril protein degradation and promoting gel weakening. This was consistent with the decrease in G′, G″, and the increase in tan δ as CP increased between 40 °C and 50 °C (Figure 1a,c,e). Nakashima et al. [58] reported that proteolytic enzymes were found in the alimentary canal of crickets and their activities were similar to trypsin with an optimal temperature of 50 °C. It might be suggested that exogenous proteolytic enzymes from CP could stimulate the breakdown of myofibrillar proteins, particularly the myosin heavy chain. The cumulative TCA-soluble peptide content in the resultant gel added with CP can also be increased by the presence of endogenous peptides in the CP. However, other parameters had to be considered carefully in regards to gel properties. In contrast, the presence of SPI exhibited a significant decrease in TCA-soluble peptide content compared to control and CP samples, and the lowest was found in the gel added with 1% SPI (*p* < 0.05). This was in line with the marginal drop in G′, G″, and reduction in tan δ between 40 °C and 50 °C (Figure 1b,d,f). Luo et al. [34] reported that SPI contains some inhibitors of aquatic protease. It could inhibit the weakening of the gel in silver carp surimi, which is a major contribution of the breakdown of myofibrillar proteins during heating. Therefore, the effect of SPI on the reduction in mackerel surimi degradation may be due to a reduction in the level of surimi protein caused by the increase in the concentration of SPI; additionally, SPI demonstrated inhibitory activity toward gel weakening inducing proteinase from mackerel. Nevertheless, another reason causing the increase in TCA-soluble peptide content with the increase in the protein additives might be the high number of free small peptides presented in the CP and SPI. This was not associated with enzymatic degradation occurring in the surimi gel, and this could also explain why the TCA-soluble peptide content increased.

#### 3.2.2. Breaking Force, Deformation, and Gel Strength

Gel strength is defined as the product of breaking force and deformation, providing the evidence of surimi products in grading [45]. Deformation and breaking force are reflected in the elasticity and gel strength of mackerel surimi gels, respectively. Figure 2 shows that the results of the gel properties were practically in agreement with those of the dynamic rheological properties (Figure 1). Surimi gel-breaking force and deformation were correlated with the rheological parameters G’ and G”. For gels containing both protein additives, a positive correlation between breaking force or deformation and G’ or G” (at 90 °C) was noted, with an R^2^ range from 0.6737 to 0.9807. R^2^ values for the SPI treatment were 0.9016, 0.8726, 0.7719, and 0.6814, respectively, for correlations between breaking force vs. G’, breaking force vs. G”, deformation vs. G’, and deformation vs. G”. The R^2^ of each correlation for the CP treatment were 0.7151, 0.6737, 0.9807, and 0.9598, respectively, as compared to the SPI treatment. The addition of CP and SPI with different levels had diverse impacts on the property of the mackerel surimi gels. The breaking force and deformation of the surimi gels increased significantly as CP and SPI increased (*p* < 0.05). Compared between the gels added with CP and SPI, the breaking force and deformation of the SPI samples were markedly higher than those of the CP samples at each concentration (*p* < 0.05). At a concentration of 5%, the gels added with CP and SPI reached their maximum breaking forces of 21.81 g and 60.87 g, respectively (Figure 2b), while the deformation was estimated at 5 mm and 8.5 mm, respectively (Figure 2c). Consequently, the gel strength (breaking force × deformation) at this level of SPI incorporation was 536.27 g.mm, 8.6 times greater than that in the control gel; meanwhile, CP incorporation at the same level was about 117.35 g.mm, just 1.9 times higher than that in the control gel (Figure 2d). These results suggest that CP and SPI can improve the gel strength of mackerel surimi. For SPI, it is speculated that incorporation of SPI into surimi could act as a filled-gel system, which could interconnect with surimi proteins to create a more robust system, making the gel network denser and eventually showing an increase in the gel strength. 

The heating process could facilitate the unfolding of the native form of SPI and the exposure of buried non-polar groups. As a consequence, the protein–protein interaction is strengthened by various chemical interactions, particularly hydrophobic associations, which were the dominant bonds that increased with the increase in SPI concentration (Table 1). Wang et al. [33] suggested that hydrophobic interactions played the most important role in stabilizing SPI-added myofibrillar protein gels, which were advantageous in improving the G′ and textural properties. Thus, SPI might encourage the interaction between myofibrillar proteins, resulting in a final gel structure that is more robust and elastic. Additionally, the protease inhibitors from SPI were linked to decreased surimi gel autolysis, enhancing the gel strength of the surimi gel [59]. For CP, the term of physical space-filling effect might not be the main reason for explaining the strengthening of CP incorporated in surimi gel compared to the SPI addition. Exposure of reactive groups in the CP during heating might facilitate the formation of and strengthen the resulting gel. In addition, CP has been the source of nutritional values that contain not only high amounts of protein but also minerals and bioactive compounds that may have the capacity to enhance the gel properties. Kosečková et al. [60] reported that CP (*A. domesticus*) contained several minerals, among which calcium was the dominant, estimated at 176–256 mg/100 g dry weight, and chitin and chitosan were also found in CP [61]. Calcium and chitosan may participate in the gel formation due to activation of the transglutaminase (TGase) enzyme. Thus, potential mechanisms for boosting the gel strength of surimi containing CP include the activation of endogenous TGase by calcium and the gel-strengthening effect of chitosan. As documented by Benjakul et al. [62], the addition of chitin/chitosan improved the breaking force and deformation of gels prepared from barred garfish (*Hemiramphus far*) surimi. However, the presence of protease enzymes in CP could adversely affect the gelation after setting the gel, which is why the gels added with CP had a lower gel strength than the gel added with SPI.

#### 3.2.3. EMC

The EMC typically serves as an indicator for the water-holding capacity (WHC) of the surimi gel. Higher WHC is associated with lower expressible drip. As shown in Figure 2e, the control gel showed the highest EMC (*p* < 0.05). The higher EMC for the control gel was probably due to the inadequate gel network caused by the low gel-forming ability of mackerel surimi [63]. The EMC of surimi gels added with CP and SPI decreased significantly with higher concentration (*p* < 0.05). When CP and SPI were added up to 3–5%, the EMC of the gel tended to be stable, which decreased by approximately 22.65% and 51.94%, respectively, compared to the control gel. This result demonstrated that water may be retained in the matrix of the protein network or bound to the functional groups of CP or SPI to form a firmer network structure resulting from the incorporation of CP and SPI. This increased the amount of water that was trapped in the gel matrix and raised the WHC of the surimi gel. The lower EMC showed that more desirable physical water-trapping and chemical–water interactions took place in the protein matrix [18]. Generally, the lower EMC coincides with the increased gel strength (Figure 2d). The effect was linked to the increase in the breaking force and gel strength of the resulting gels when CP and SPI were added (Figure 1a,c). When compared at the same level added, it was found that SPI markedly reduced the EMC more than CP at each concentration (*p* < 0.05). From the result, SPI may help create a stronger network than CP that is capable of retaining more water in the gel network. Sze-Tao and Sathe [64] stated that SPI mainly contains water-insoluble globulins, resulting in a high swelling property (high viscosity). Additionally, the more compact and uniform gel structure brought about by SPI and the increased intermolecular interactions that result from heating could have a more beneficial effect on the water entrapment in the gel network. The intrinsic water absorption properties of each protein, whereby they interact with myosin to form a tighter network to bind water, account for the variations in the effects of the addition of two types of protein supplement on the EMC of mackerel surimi gels. The degree of protein denaturation that results from heating varies, however, and this results in a variety of three-dimensional network structures and moisture retention [65].

#### 3.2.4. Whiteness and Appearance

The whiteness of the mackerel surimi gel added with CP or SPI gradually decreased with increasing levels (*p* < 0.05) (Figure 2f,g). Adding 5% CP and 5% SPI to the mackerel surimi gels decreased their whiteness by 5.8% and 20.7%, respectively. The reason is that the brownish and yellowish color of CP and SPI, respectively, greatly diminishes the whiteness of mackerel surimi gels. Mi et al. [32] reported that the light yellow of the SPI was detrimental to the whiteness of Zhikong scallop adductor muscle gel; Meanwhile, the brown color of the CP contributed to the substantial dark color of bread and cookies enriched with CP, according to Bawa et al. [66]. Similar to previous reports, some gel strengtheners have a detrimental impacts on the color of the gel. Wang et al. [67] found that the high concentration of chickpea protein isolate and peanut protein isolate decreased the whiteness of hairtail (*Trichiurus haumela*) myosin gels. Arsyad et al. [14] reported that the addition of olive leaf powder to red sea bream surimi led to a decrease in whiteness. The content and types of exogenous additives added could influence the color characteristics of fish muscle protein gels [12,68,69]. Whiteness is one of the most important sensory parameters to judge the quality of surimi products. Therefore, in order to retain the overall aspect, CP and SPI should be used at the appropriate concentrations.

### 3.3. TPA

TPA is an assessment index of food texture, mainly compressed twice by simulating the chewing action of the human mouth to obtain parameters corresponding to consumer desirability [43]. The TPA parameters of gels from mackerel surimi added with CP and SPI at various levels are shown in Table 1. From the result, the addition of 5% CP could significantly increase the hardness, springiness, cohesiveness, chewiness, and gumminess of the mackerel surimi gel compared to the control sample (*p* < 0.05). However, when comparing at the same level of protein additives, it was found that all TPA parameters of surimi gel added with SPI were markedly higher than those of CP-added gels (*p* < 0.05). At the incorporation of 5% SPI, the surimi gel demonstrated the highest hardness (60.85 g), which was six times harder compared to the control gel (10.53 g), whereas the CP incorporated at the same level was slightly higher in hardness (14.63 g). This was consistent with the gels’ breaking force (Figure 2b). Pietrasik et al. [70] reported that 2% commercial SPI improved the hardness of pork-meat gel. However, due to the lower protein content in powder and the less effective role in modifying the properties of the gel matrix, CP showed a lesser gel-strengthening impact than SPI. The results confirmed that the addition of CP and SPI to surimi could enhance the gel strength. Similar results were also observed for gumminess and chewiness. The results indicated that SPI can facilitate a harder elastic complex gel than CP with a higher gumminess and chewiness which was consistent with the increase in gel strength (Figure 2c). The chewiness and gumminess measurements were usually based on the hardness [18]. When compared to the control, the addition of 3–5% SPI considerably improved the surimi gel’s cohesiveness and springiness, but CP needed to be added up to 5% to obtain the same results. This result was coincident with the gel deformation value (Figure 2c). As a consequence, both CP and SPI, particularly at 5% concentration, may contribute to the cross-linking of proteins in various ways, with multiple interactions of unfolded proteins between protein additives and myofibrillar proteins promoting the filling or binding effect and thereby strengthening the gel network. This could lead to the different TPA characteristics of the gels.

### 3.4. Molecular Driving Forces

The solubility of protein gels in different solvents reflects different molecular reactions in the gel structure. The chemical substances in solutions can break the gels’ corresponding chemical bonds, increasing solubility [71]. As shown in Table 2, the hydrophobic interaction was remarkably higher than those of other chemical forces in all samples (*p* < 0.05). This revealed that hydrophobic interaction was the main driving force in surimi gels, maintaining the structure of the network. With an increase in the SPI level, the hydrophobic interaction index increased markedly, whereas it was needed to add up to 5% of CP to significantly increase the hydrophobic interaction in comparison to the control sample (*p* < 0.05). The result of hydrophobic interaction was in a trend similar to that of the hydrogen bond index. The hydrogen bond content has a certain connection with the WHC. A small number of exogenous additives can form hydrogen bonds with free water [72]. It should be noted that the result was in agreement with the gel strength, texture, and expressible moisture changes. However, the addition of 5% CP resulted in a rise in the disulfide bond content, which could, in part, have intensified the effects on the protein gel. This phenomenon was caused by the fact that small levels of CP could encourage the covalent cross-linking of myosin. However, the proportion of disulfide bonds in the gels tended to decrease as the concentration of SPI increased. So, the addition of SPI inhibited the formation of disulfide bond and this bond was not the primary covalent bond reinforcing the gel network. Peng and Nielsen [73] reported that the presence of β-conglycinin in SPI decreased the aggregation of myosin heavy chains between 50 °C and 100 °C. The hydrophobic association and hydrogen bonds were largely attributed to the stronger interaction between SPI and muscle proteins upon heating, whereas the disulfide linkages did not significantly contribute to the improvement in gel strength of porcine myofibrillar protein [59]. O’Kane et al. [74] also noted that hydrophobic and hydrogen bonds had a major role in the legumin proteins’ ability to form networks; however, disulfide bonds played a minor one. The ionic bonding of the surimi gel treated with CP increased as the concentration rose in comparison to the control gel (*p* < 0.05). On the contrary, the addition of SPI showed the highest ionic bonds in the gel added with 1% SPI concentration and the ionic bonds vanished when the level of SPI increased (*p* < 0.05). An increase in ionic bonds suggested that the CP might change the charge distribution sites of amino acids. In denaturing proteins, both favorable and unfavorable electrostatic interactions may take place simultaneously, and the differences in ionic bonding in mixed gels may be related to various denatured processes of protein and peptide. The presence of chitosan in CP might induce the electrostatic interactions as a result of the increase in the ionic bonds. Alishahi and Aïder [75] stated that electrostatic interactions and hydrogen bonding played a major role in stabilizing the association between chitosan and salt-soluble muscle proteins. Huang et al. [76] stated that, in myofibril protein isolate-SPI, the ionic interactions weakened with rising temperature, especially when the temperature exceeded 65 °C. On the other hand, due to charge anisotropy, the strong ionic interaction between nearby protein molecules may be explained by the positively and negatively charged amino acid residues. Liu et al. [77] demonstrated that hydrogen bonds and ionic bonds had a minor contribution to the surimi gel structure but hydrophobic interaction was the most important chemical interaction to maintain the surimi gel network. From the relative content shown in Table 2, it implies that hydrogen bonds and ionic interactions may not be the primary driving forces in maintaining gel stability [32,78]. In contrast, modifications in hydrophobic interactions may play a significant role in the variations in mixed gel structure.

### 3.5. SDS-PAGE Protein Patterns

**Figure 3:**
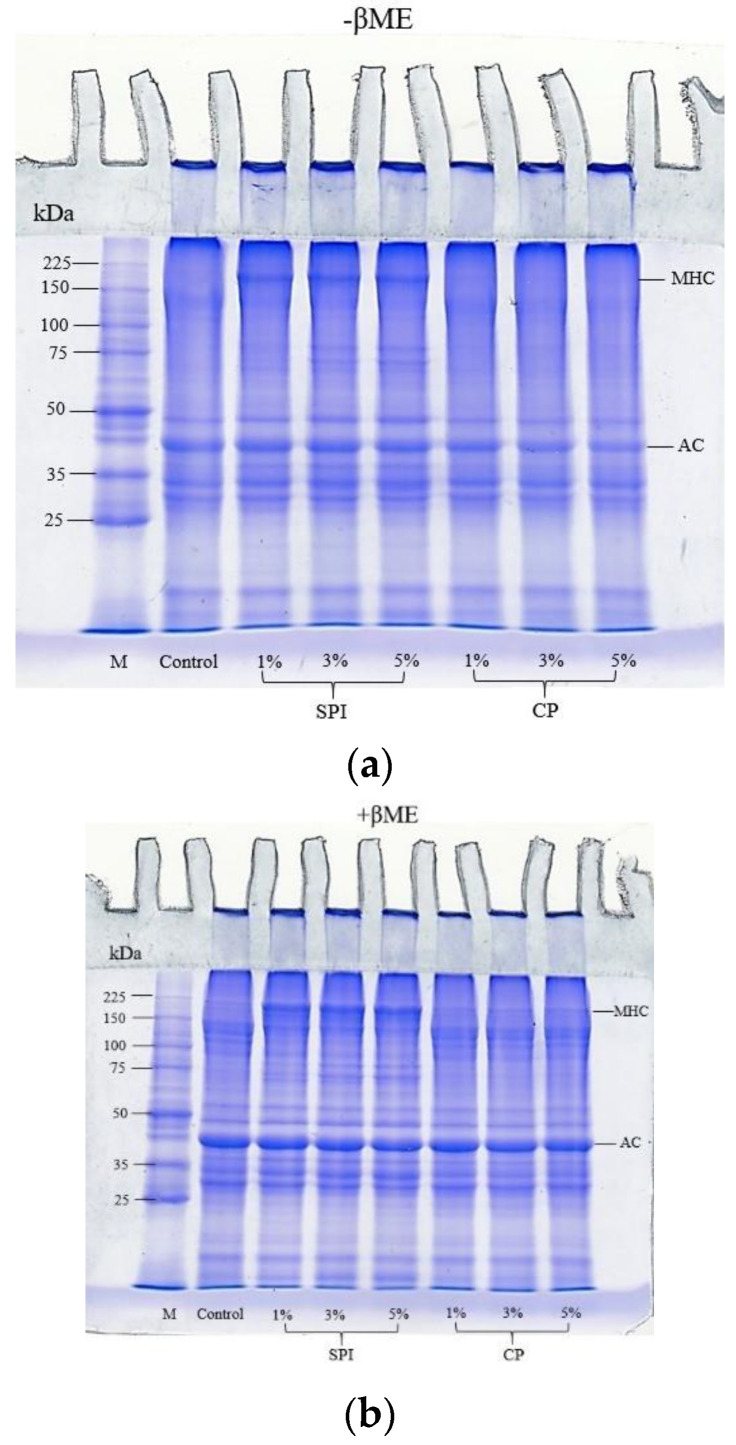
Effects of the addition of cricket powder (CP) and soy protein isolate (SPI) at different concentrations on protein patterns of mackerel surimi gels under non-reducing (**a**) and reducing conditions (**b**). MHC, myosin heavy chain; AC, actin; M, protein markers; βME, β-mercaptoethanol (a reducing agent). Samples (15 μg protein) were loaded onto the polyacrylamide gel comprising a 10% running gel and a 4% stacking gel and subjected to electrophoresis at a constant current of 15 mA per gel.

The protein patterns of surimi gels with and without the addition of CP and SPI at different levels are shown in Figure 3. Myosin heavy chain (MHC) and actin were found to be the main proteins of the surimi paste. All samples without βME showed the presence of covalent bonds because a thick band indicating high-molecular-weight protein complexes was polymerized and visible at the top of the stacking gel. Inside the control gels, the MHC disappeared entirely. The cross-linking of MHC through covalent bonds can promote the formation of surimi gels, resulting in a fainting effect of the MHC band intensity (Figure 3a). In the presence of a reducing agent, such as βME, the majority of polymerized proteins can be separated. Although some of the protein aggregates were still present in the stacking gel, this indicates the existence of additional bonds, such as hydrophobic interactions (Figure 3b). Compared to the control, the intensity of the MHC band did not show significant changes at all levels of CP, but the intensity of the actin band appeared to decrease with increasing CP, particularly at a concentration of 5% (Figure 3a). This result suggests that a large amount of CP may implicate the participation of actin in the generation of non-disulfide covalent bonds, resulting in a synergistic role in the gelation of myosin, which consequently increases the rigidity of the gel. Unexpectedly, the high intensity band of MHC was still found in the surimi gel with the SPI added. It was suggested that some MHC did not polymerize to form the gel by disulfide bonds. This could be because the main interaction between SPI and myosin was non-covalent bonds, particularly hydrophobic interactions and hydrogen bonds (Table 2). Therefore, disulfide linkages were not a key driving force for the improvement in gel strength induced by SPI. Hydrophobic interactions and hydrogen bonding were found to be crucial in the development of heat-induced myofibrillar protein–SPI gels, according to Wang et al. [33]. For all gel samples with SPI added, a gradual increase in the intensity of the protein bands around 50–75 kDa was observed as the SPI level raised, which may be due to the presence of the 7S protein subunit of SPI. This protein might interfere with the crosslink of disulfide bonds of MHC to form the gel. According to Feng and Xiong [79], 7S globulins negatively affected the self-aggregation of MHC. Furthermore, the addition of SPI could decrease the number of protein bands and the intensity of the bands ranged from 150 to 75 kDa (Figure 3b), indicating that proteolytic degradation could be inhibited during heating, as indicated by the lower contents of TCA-soluble peptides (Figure 2a). Thus, this reason could be also used to explain why the surimi gel with SPI added had significantly improved textural properties (Figure 2 and Table 1).

### 3.6. FTIR Spectroscopy of Gels

**Figure 4:**
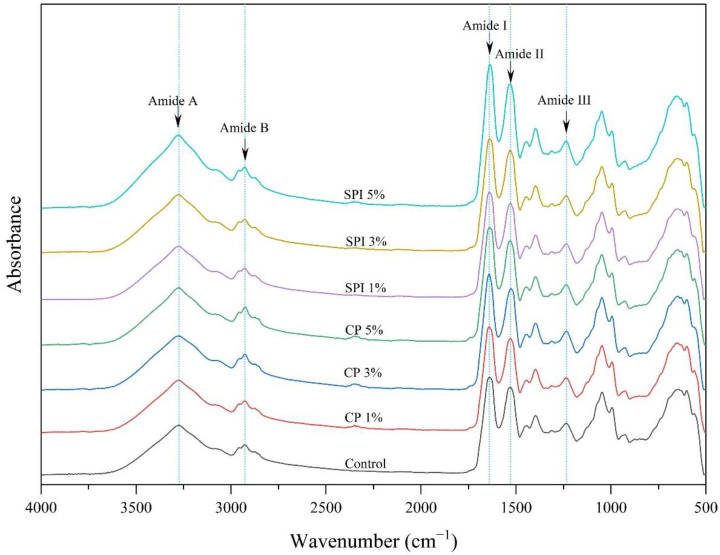
FTIR spectra of surimi gels with the addition of cricket powder (CP) and soy protein isolate (SPI) at different levels.

FTIR spectroscopy is a helpful technique to investigate the structural changes of proteins [80]. As presented in Figure 4, the infrared spectra of mackerel surimi gels with the addition of CP and SPI at different levels were scanned, ranging from 4000 to 500 cm^−1^. During heating to produce surimi gel, protein solubilization by salt resulted in a decrease in α-helix structures and an increase in β-sheet structures [81]. The main and typical peaks were observed in the infrared range of 1200, 1500, 1700, and 3100–3300 cm^−1^, respectively, which correspond to amides III, II, I, and amides B and A vibrational states in the protein structure [82].

In Figure 4, the similar spectra were noticeable in all samples in which the primary bands in the FTIR spectra were recorded as amide I, amide II, amide III, amide A, and amide B. At 1640 cm^−1^, the amide I band, a major peak in the spectra of all gels arising from C=O stretching vibration and frequently employed as probes for protein conformation, was found, indicating a predominance of β-sheet as well as a more stable structure [83]. The N-H bending vibration and the C-N stretching vibration are responsible for the amide II band, which was found at 1529 cm^−1^. Amide III (C-N stretching vibration and N-H deformation) is detectable at 1235 cm^−1^. The N-H stretching vibration of amide A was found at 3274 cm^−1^ and the C-H stretching vibrations of –CH_2_ group of amide B were observed at 2927 cm^−1^. The addition of CP or SPI at all levels into the surimi to formulate the gels did not change the FTIR spectra and did not produce new absorption peaks, indicating that the same protein functional groups were present in the gel structure.

### 3.7. Microstructure 

**Figure 5:**
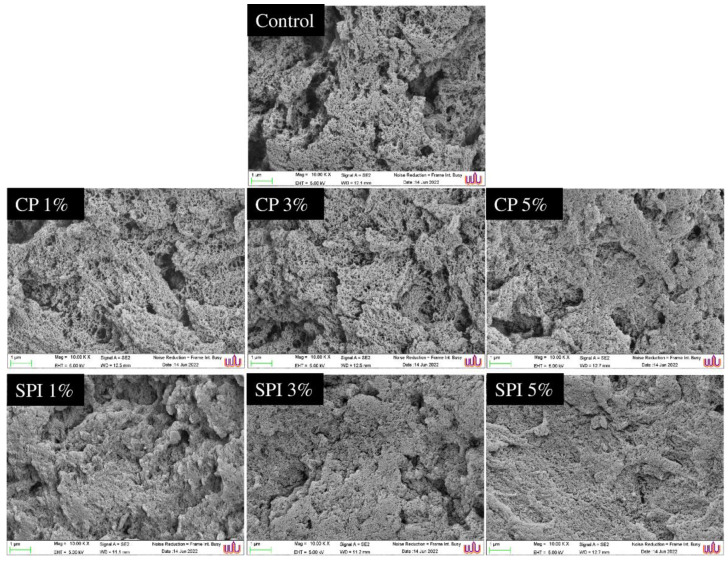
Effects of the addition of cricket powder (CP) and soy protein isolate (SPI) at different levels on the microstructure of surimi gels. (Magnification: 10,000×, EHT: 3 kV).

The SEM images of mackerel surimi gels containing CP or SPI at various concentrations are shown in Figure 5. The control gel without CP and SPI had a loose and coarse microstructure with a large number of cavities, which was consistent with the minimum gel strength and EMC (Figure 2d,e). After the addition of SPI, the gel microstructure became smoother and denser as the concentration increased. A gel with 5% SPI was found as the most ordered network structure, which was related to the highest G′ (Figure 1a), maximum gel strength (Figure 2d) and the lowest EMC (Figure 2e). This might be because after mixing, the SPI molecules scattered throughout the network structure. SPI absorbed the ambient water during heating, had repellent and supporting effects on the protein network, filled gaps between myofibrillar proteins, encouraged direct, limited adhesion of proteins, made the protein gel network more compact and orderly, and enhanced the protein gel-network’s structural integrity [84]. The presence of 5% CP seemed to promote more compact and uniform gel than 1 and 3% CP. Similar reasons could also be used to explain the microstructure of the gel with CP added. However, the chemical interaction of SPI and CP were different; thus, leading to a different gel microstructure. Notably, when compared to the gel containing CP, the mackerel surimi gels containing SPI exhibited a more compact and ordered protein network with fewer holes. It might support our claim that the gel network structure was greatly influenced by the filling impact of protein additives and the interaction of foreign proteins with the continuous phase myosin matrix. As a result, the addition of CP or SPI in the right proportion can encourage molecular cross-linking during the creation of mackerel surimi gel and enhance the gelling capabilities (Figure 2 and Table 1). 

### 3.8. Sensory Properties

**Figure 6:**
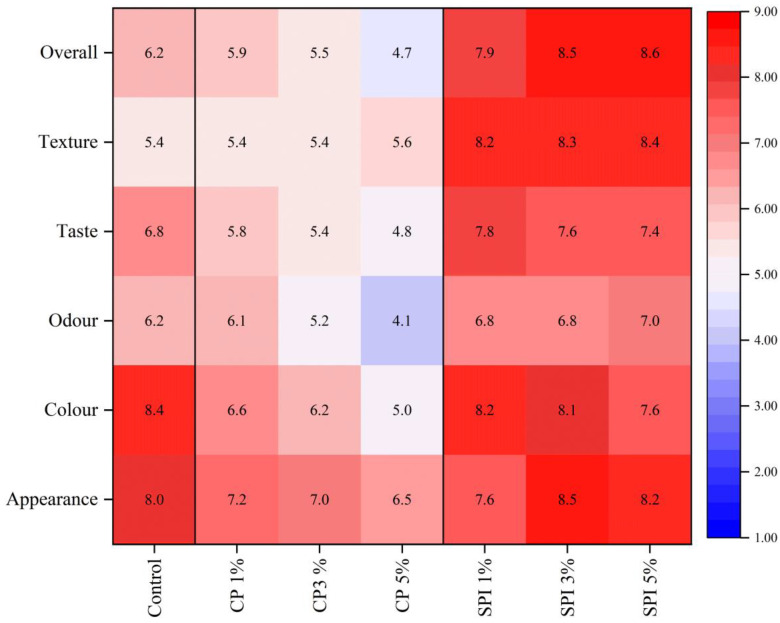
Heatmap of sensory properties of surimi gels with the addition of cricket powder (CP) and soy protein isolate (SPI) at different levels.

To better evaluate the mackerel surimi gel added with CP and SPI at different concentrations, sensory evaluations were performed using a nine-point hedonic scale. The parameters of sensory properties are: appearance; odor; color; taste; texture; and overall acceptability. As shown in Figure 6, SPI had a promoting effect on all of the parameters of the surimi gel with a higher score except for the color parameter compared to the control gel. The results indicated that whiter gels were more preferred. The texture score of surimi gels was dramatically raised by the addition of SPI. This was in agreement with the better texture profiles, including hardness, springiness, cohesiveness, chewiness, and gumminess, with the increase in SPI level (Table 1). In contrast, the addition of CP had a detrimental effect on several parameters of mackerel surimi gel. The decrease in odor, color, and taste scores resulting in the lower of overall acceptability as the concentration of CP increased. However, the marginal increase in the texture score was found at a 5% concentration of CP. Thus, the addition of CP at a suitable level could enhance the surimi gel products, but color, odor, and taste were limited to being used in certain gel products. 

### 3.9. Lipid Oxidation

**Figure 7:**
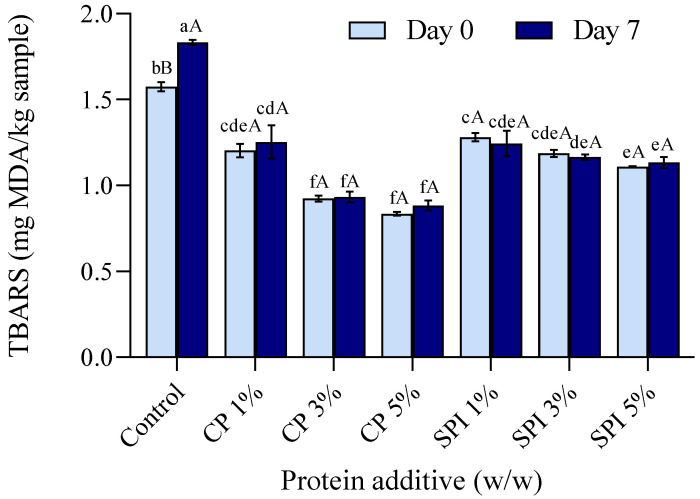
Changes of lipid oxidation assessed by thiobarbituric acid reactive substances (TBARS) of mackerel surimi gels prepared using the addition of cricket powder (CP) and soy protein isolate (SPI) at Day 0 and Day 7 of refrigerated storage (4 °C). Bars represent standard deviation from triplicate determinations. Different lowercase letters across all samples and different uppercase letters between storage times at the same level of additive indicate significant differences (*p* < 0.05).

Lipid oxidation has a negative impact on the quality of fishery products, which results in an off-flavor and off-odor [85]. The content of TBARS, expressed in mg MDA equivalent/kg of sample, is widely considered as an index parameter for measuring secondary lipid oxidation products [86]. As shown in Figure 7, the highest TBARS were found in the control gel. Fish muscle, particularly that of dark-fleshed fish such as mackerel, contains iron bound to protein, which could be released during heat-induced gelation. Free iron may have acted as a pro-oxidant, accelerating the oxidation of lipids [87]. However, when CP and SPI were added, the TBARS tended to decrease significantly and the lowest TBARS were observed in surimi gel added with 3 and 5% CP (*p* < 0.05). When the surimi gels were kept at 4 °C for 7 days, the TBARS of the control sample increased markedly compared to Day 0 (*p* < 0.05). However, the other samples were not different in TBARS after being kept for 7 days. This result showed that the addition of CP and SPI could delay the lipid oxidation of mackerel surimi gel during storage at 4 °C for 7 days, indicating that CP and SPI have some antioxidant effect against lipid oxidation in mackerel surimi. Hayes et al. [88] stated that the main antioxidants in soy products included amino acids, peptides, isoflavone glycosides, and their aglycones. Pork patties with 2% SPI added and held at 4 °C had lower TBARS contents than the control, according to Peña-Ramos and Xiong [89]. A valuable source of bioactive peptides with antioxidant action through inhibiting the activities of lipoxygenase and cyclooxygenase-2, considerable antiradical activity, and the ability to chelate iron ions has been discovered in edible insects, especially crickets [90]. Furthermore, chitin and chitosan are abundant in crickets; in particular, chitosan has antioxidant properties. Additionally, chitosan was found to greatly reduce lipid oxidation, according to several studies. According to Mohan et al. [91], the main amino groups in chitosan formed a stable fluoresphere with volatile aldehydes that are produced from the breakdown of lipids during oxidation, which may be related to the antioxidation mechanism of chitosan. The Maillard reaction products that were initially discovered in CP and SPI as well as those newly created during thermal gelation may also be an additional source of antioxidants that can work in concert to delay the oxidation of the lipids in the mackerel surimi during refrigeration. Antioxidative Maillard reaction products (MRPs) have been created using both soy proteins [92] and edible insect proteins [93].

## 4. Conclusions

The addition of SPI improved the texture, WHC, and sensory qualities of mackerel surimi gel while slightly reducing the whiteness. For the CP, up to 5% had to be added to significantly improve the gel characteristics of mackerel surimi compared to the control gel. The significant decrease in whiteness was also a detrimental effect of the addition of CP. The findings showed that SPI has superior gel enhancement and sensory retention abilities compared to CP. However, CP had a remarkable potential to delay lipid oxidation during storage at 4 °C for 7 days. Overall, the amount of 5% SPI or CP can be used as functional and nutritional ingredient for improving the gel-forming ability of mackerel surimi. However, CP still had a detrimental effect on customer acceptability. It is necessary to investigate further strategies for improving the sensory features of employing CP in mackerel surimi, such as combining it with other seasoning ingredients to make the final gel products more palatable.

## Figures and Tables

**Table 1:** Effects of the addition of cricket powder (CP) and soy protein isolate (SPI) on the texture profile analysis (TPA) parameters of mackerel surimi gels.

Samples	Levels (%)	Texture Profile Analysis Parameters				
		Hardness (N)	Springiness (mm)	Cohesiveness	Chewiness (N)	Gumminess (N)
Control	-	10.53 ± 0.73 ^e^	6.68 ± 0.27 ^d^	0.13 ± 0.01 ^d^	9.08 ± 0.92 ^e^	1.42 ± 0.13 ^e^
CP	1	10.67 ± 0.72 ^eB^	6.43 ± 0.08 ^dB^	0.13 ± 0.00 ^dB^	9.11 ± 0.88 ^eB^	1.44 ± 0.11 ^eB^
	3	12.28 ± 0.94 ^eB^	6.58 ± 0.18 ^dB^	0.13 ± 0.02 ^dB^	10.70 ± 1.48 ^eB^	1.62 ± 0.18 ^deB^
	5	14.63 ± 1.47 ^dB^	8.48 ± 0.12 ^cB^	0.14 ± 0.01 ^cB^	17.70 ± 3.08 ^dB^	1.87 ± 0.04 ^dB^
SPI	1	44.22 ± 4.91 ^cA^	10.09 ± 0.02 ^bA^	0.39 ± 0.01 ^bA^	172.05 ± 17.09 ^cA^	17.05 ± 1.80 ^cA^
	3	52.52 ± 5.00 ^bA^	10.36 ± 0.07 ^aA^	0.41 ± 0.01 ^aA^	223.22 ± 17.17 ^bA^	21.48 ± 1.75 ^bA^
	5	60.86 ± 3.03 ^aA^	10.32 ± 0.07 ^aA^	0.41 ± 0.01 ^aA^	255.32 ± 13.90 ^aA^	24.80 ± 1.38 ^aA^

Different lowercase superscripts in the same column indicate significant differences (*p* < 0.05). Different uppercase superscripts in the same column under the same level of CP and SPI indicate significant differences (*p* < 0.05). Data are expressed as mean ± standard deviation, *n* = 6.

**Table 2:** Effects of the addition of cricket powder (CP) and soy protein isolate (SPI) on molecular driving forces of mackerel surimi gels.

Samples	Level (%)	Soluble Protein Contents (g/L)			
		Ionic Bonds	Hydrogen Bonds	Hydrophobic Interactions	Disulfide Bonds
Control	-	0.64 ± 0.01 ^c^	0.34 ± 0.01 ^c^	2.44 ± 0.06 ^e^	1.51 ± 0.02 ^b^
CP	1	0.62 ± 0.01 ^cdB^	0.37 ± 0.03 ^cA^	2.41 ± 0.01 ^eB^	1.26 ± 0.07 ^cA^
	3	0.64 ± 0.04 ^cA^	0.35 ± 0.04 ^cB^	2.89 ± 0.06 ^dB^	1.18 ± 0.05 ^cA^
	5	0.80 ± 0.01 ^bA^	0.47 ± 0.01 ^bB^	2.84 ± 0.11 ^dB^	1.77 ± 0.10 ^aA^
SPI	1	0.85 ± 0.01 ^aA^	0.35 ± 0.01 ^cA^	4.06 ± 0.06 ^cA^	0.87 ± 0.07 ^dB^
	3	0.60 ± 0.03 ^dA^	0.49 ± 0.02 ^bA^	5.55 ± 0.15 ^bA^	0.73 ± 0.10 ^dB^
	5	0.50 ± 0.01 ^eB^	0.56 ± 0.02 ^aA^	6.88 ± 0.08 ^aA^	0.36 ± 0.08 ^eB^

Different lowercase superscripts in the same column indicate significant differences (*p* < 0.05). Different uppercase superscripts in the same column under the same level of CP and SPI indicate significant differences (*p* < 0.05). Data are expressed as mean ± standard deviation, *n* = 3.

## Data Availability

Data is contained within the article.
